# Validation of deep learning-based computer-aided detection software use for interpretation of pulmonary abnormalities on chest radiographs and examination of factors that influence readers’ performance and final diagnosis

**DOI:** 10.1007/s11604-022-01330-w

**Published:** 2022-09-19

**Authors:** Naoki Toda, Masahiro Hashimoto, Yu Iwabuchi, Misa Nagasaka, Ryo Takeshita, Minoru Yamada, Yoshitake Yamada, Masahiro Jinzaki

**Affiliations:** Department of Radiology, Keio University School of Medicine, 35 Shinanomachi, Shinjuku-ku, Tokyo, 160-8582 Japan

**Keywords:** Chest radiography, Computer-aided detection, Deep learning, Pulmonary lesions, Multicenter study

## Abstract

**Purpose:**

To evaluate the performance of a deep learning-based computer-aided detection (CAD) software for detecting pulmonary nodules, masses, and consolidation on chest radiographs (CRs) and to examine the effect of readers’ experience and data characteristics on the sensitivity and final diagnosis.

**Materials and methods:**

The CRs of 453 patients were retrospectively selected from two institutions. Among these CRs, 60 images with abnormal findings (pulmonary nodules, masses, and consolidation) and 140 without abnormal findings were randomly selected for sequential observer-performance testing. In the test, 12 readers (three radiologists, three pulmonologists, three non-pulmonology physicians, and three junior residents) interpreted 200 images with and without CAD, and the findings were compared. Weighted alternative free-response receiver operating characteristic (wAFROC) figure of merit (FOM) was used to analyze observer performance. The lesions that readers initially missed but CAD detected were stratified by anatomic location and degree of subtlety, and the adoption rate was calculated. Fisher’s exact test was used for comparison.

**Results:**

The mean wAFROC FOM score of the 12 readers significantly improved from 0.746 to 0.810 with software assistance (*P* = 0.007). In the reader group with < 6 years of experience, the mean FOM score significantly improved from 0.680 to 0.779 (*P* = 0.011), while that in the reader group with ≥ 6 years of experience increased from 0.811 to 0.841 (*P* = 0.12). The sensitivity of the CAD software and the adoption rate for the lesions with subtlety level 2 or 3 (obscure) lesions were significantly lower than for level 4 or 5 (distinct) lesions (50% vs. 93%, *P* < 0.001; and 55% vs. 74%, *P* = 0.04, respectively).

**Conclusion:**

CAD software use improved doctors’ performance in detecting nodules/masses and consolidation on CRs, particularly for non-expert doctors, by preventing doctors from missing distinct lesions rather than helping them to detect obscure lesions.

## Introduction

Chest radiography is a commonly used medical imaging and diagnostic technique for initial screening of patients due to its low cost and easy accessibility [1]. It plays an important role in detecting lung diseases such as lung cancer and tuberculosis [2, 3].

However, accurate interpretation of chest radiographs (CRs) can occasionally be challenging for doctors. Approximately 90% of missed lung cancer cases involve CR assessments [4], and the miss rate of lung cancers on CRs is reportedly 19–22% [5, 6]. The characteristics of abnormal lesions, such as size, conspicuity, and location, influence the detection accuracy [4, 7]. Reader proficiency is another important factor. While expert observers establish specific scanning patterns for radiographs, non-expert observers generally search without order on the radiograph, and this can cause them to overlook obscure abnormal findings [4]. In Japan, doctors who do not specialize in pulmonology often read CRs in daily practice. Approximately 50% of the doctors who interpret CRs for patients screening are not lung disease experts, such as non-pulmonology physicians [8], and this may reduce the abnormality detection rate on CRs. Thus, there is a demand for detection tools for non-experts.

In recent years, Computer-aided detection (CAD) systems that use deep learning algorithms have been developed [9, 10]. Some studies have shown that observer performance for detection of abnormal thoracic lesions with CAD is significantly better than without CAD [11, 12]. These studies used algorithms developed by researchers from scratch, but few studies have used software products developed by vendors. Some CAD software packages for CRs are already commercially available, and their diagnostic performance has been reported (e.g., EIRL X-ray Lung nodule, Lpixel, Tokyo, Japan). However, few studies have analyzed data characteristics that affected improvement of readers’ performance and final diagnosis with CAD.

This study aimed to compare doctors' performance in interpreting CRs with and without CAD. We also examined the effect of readers’ experience and data characteristics on detection of abnormal pulmonary lesions with CAD. Lung nodules, masses, and consolidation on CRs were targeted because the CAD software used in this study was designed to detect these lesions.

## Materials and methods

This retrospective multicenter study was approved by the institutional review board, and anonymized data were shared through a data-sharing agreement between the institutions. The requirement for written informed consent was waived because the data were collected retrospectively. This study was supported by Konica Minolta.

### Data collection

Anonymized CRs (posteroanterior view) of 453 patients were retrospectively selected from two institutions in Japan. Only patients who were over the age of 19 years were included. Institution A, a university hospital, supplied the data of 238 patients who presented for physical examination between January 2012 and December 2019. Institution B, a health screening center, supplied the data of 215 patients who had routine health screening between January 2016 and December 2018. One CR image was acquired from each patient; thus, 453 images were collected. The images were acquired using Aero DR system (Konica Minolta, Tokyo, Japan) or FUJIFILM DR PRELIO U(Fujifilm, Tokyo, Japan)with 120–130 kVp tube voltage, 1–8mAs tube current time product. The exclusion criterion was poor image quality, for which no CR was excluded. Pulmonary nodules/masses and consolidation on the CRs were considered abnormal findings, while extrapulmonary abnormal findings, such as cardiomegaly and rib fractures, were considered normal for the purpose of this study. Nodules and masses were defined as focal lung opacities with smooth border, measuring  ≤ 3 cm and  > 3 cm in diameter, respectively. Consolidation was defined as lung opacities apart from nodules and masses. A total of 194 images (43%) included abnormal findings, while 259 (57%) were normal. For the observer-performance test, 60 images with and 140 without abnormal findings were randomly selected. Two board-certified radiologists (with 6 and 14 years of experience) reviewed the images and recorded the area, lesion type (nodule, mass, or consolidation), and the degree of subtlety of each lesion by mutual agreement. The degree of subtlety was measured on a five-point scale as follows: level 1, extremely subtle (detection is extremely difficult); level 2, very subtle (detection is very difficult); level 3, subtle (detection is difficult); level 4, relatively obvious (detection is relatively easy); and level 5, obvious (detection is easy) [13].

### Ground truth

All images were independently reviewed by three board-certified radiologists (with 14, 25, and 33 years of experience) to establish the ground truth. The radiologists confirmed the presence of abnormal findings on the images and marked the lesion locations. The common areas annotated by at least two of the three radiologists with an intersection over union (IoU) greater than specific threshold for each finding were adopted as abnormal lesions. The thresholds for nodule/mass and consolidation were determined as 0.5 and 0.0, respectively.

### Software

CAD-Chest X-ray (Konica Minolta, Inc. and Enlitic, Inc.) software was used for this study. This software is currently commercially available in Japan (Approval No. 30300BZX00271000). This software was designed as a second-reader type CAD. It automatically detects pulmonary nodules/masses and consolidation, and marks the areas of the lesions.

### Detection performance test of the CAD software

First, a performance test of the CAD software alone (standalone test) was conducted. All 453 images in the dataset were interpreted with the software alone. Then, an observer-performance test was performed to assess whether the software would improve doctors’ performance. The test had a sequential-only design and was done in accordance with the US Food and Drug Administration guideline [14]. The CRs of the selected 200 patients were interpreted by 12 doctors, including three radiologists, 3 pulmonologists, three non-pulmonology physicians, and three junior residents with various years of experience (2–12 years). The readers were blinded to the clinical information of the patients, and the radiologists who defined the reference standard did not participate in the performance test. The test consisted of two sessions. In the first session, the readers were asked to determine whether each CR showed any nodule, mass, or consolidation. If any of these was present, the readers then marked the center of the lesion. All procedures were performed without CAD software. The readers were also asked to input a confidence score with a continuous value between zero and one for each annotation. In the second session, the readers were asked to re-evaluate every CR with the assistance of the CAD software and to modify their original decisions and confidence scores.

### Statistical analyses

The sensitivity and specificity of the CAD software for detecting pulmonary nodules, masses, and consolidation were analyzed in the standalone test. Per lesion sensitivity and patient specificity were calculated. In the observer-performance test, the detection performances of the readers with and without CAD were compared. Jackknife alternative free-response receiver operating characteristic (JAFROC) analyses were performed using R statistical software version 4.0.2 (R Project for Statistical Computing, Vienna, Austria) and RJafroc version 2.0.1. Both the readers and CRs were treated as random effects. The weighted alternative free-response receiver operating characteristic (wAFROC) figure of merit (FOM) score was used as the performance measure for the analyses. The weights were equally divided by the number of lesions. Statistical significance was evaluated using the Dorfman-Berbaum-Metz method [15]. The results were stratified according to the specialty, years of experience of the readers. The mean FOM scores with and without CAD in each group were compared. For the analysis of the sensitivity of the CAD software and the adoption rate of the lesions that readers initially missed but CAD detected, the lesions were grouped by the anatomic location and the degree of subtlety. Fisher’s exact test was used for the analyses. Statistical significance was set at *P* < 0.05.

## Results

Among the 453 images used for the Standalone test, 194 showed abnormal findings. The abnormal findings included nodules/masses in 101 (52%) images, consolidation in 91 (47%) images, and both in 2 (1%) images. 60 images with abnormal findings were selected for the observer-performance test. The abnormal findings included 36 (53%) nodules/masses and 29 (47%) consolidation. 3 images showed multiple nodules/masses and 2 images showed multiple consolidation. The demographic features of each dataset are shown in Table 1.

**Table 1:** Patients’ demographics in datasets in the standalone and observer-performance tests

	Standalone test			Observer-performance test		
	Institution A	Institution B	Total	Institution A	Institution B	Total
Age (y)^a^	62 ± 16	57 ± 12	59 ± 15	59 ± 17	57 ± 12	58 ± 15
Sex (%)						
Men	58 (139/238)	55 (119/215)	57 (258/453)	55 (56/101)	57 (56/99)	56 (112/200)
Women	42 (99/238)	45 (96/215)	43 (195/453)	45 (45/101)	43 (43/99)	44 (88/200)
Abnormal findings (%)						
Nodule (≤ 3 cm)	19 (23/123)	33 (29/87)	25 (52/210)	20 (8/40)	32 (7/22)	23 (15/65)
Mass (> 3 cm)	41 (50//123)	11 (10/87)	29 (60/210)	38 (15//40)	14 (3/22)	28 (18/65)
Consolidation	41 (50/123)	55 (48/87)	47 (98/210)	42 (17/40)	55 (12/22)	49 (32/65)

Data in parentheses are those used to calculate the percentages

^a^Mean ± standard deviation for age

### Detection performance of the CAD software

In the standalone test using 453 images, the CAD software achieved sensitivities of 83% (85/103) and 80% (74/93) for nodules/masses and consolidation, respectively. Thus, its sensitivity for detecting abnormal lesions was 81% (159/196), and its specificity was 62% (160/259).

Comparison of detection performances with and without CAD software.

**Fig. 1:**
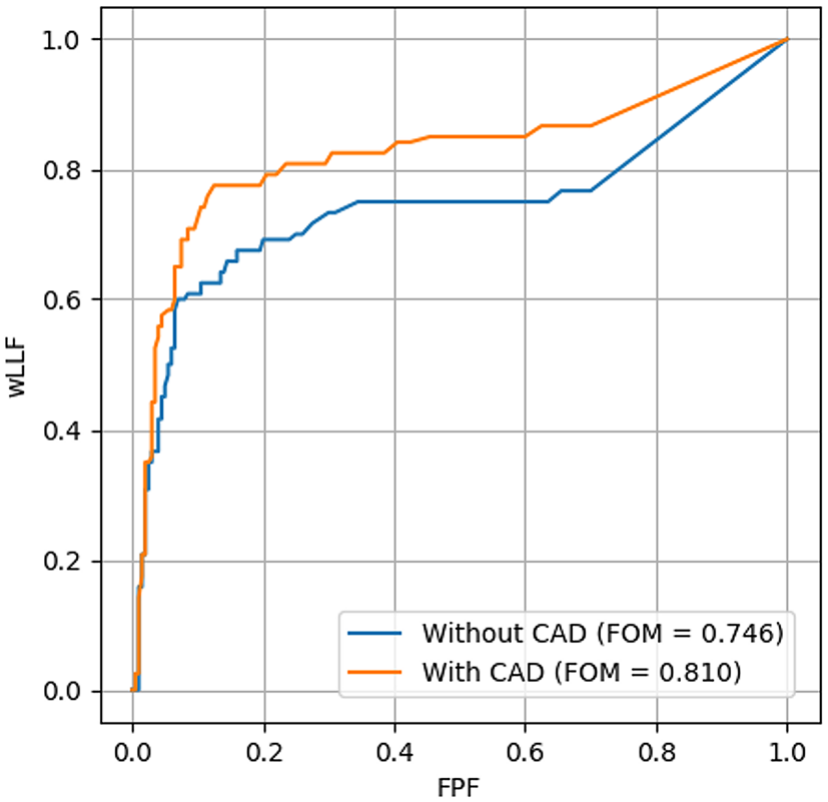
Weighted alternative free-response receiver operating characteristic curves for the observer-performance test with and without computer-aided detection (CAD) software, where the weighted lesion localization fraction (wLLF) against the false-positive fraction (FPF) has been plotted. The total result for all readers is shown. *FOM* Figure of merit

When the 200 images were used for the observer-performance test, the CAD software achieved sensitivities of 79% (26/33) and 81% (26/32) for nodules/masses and consolidation, respectively. Thus, its sensitivity for detecting abnormal lesions was 80% (52/65), and its specificity was 63% (88/140). The mean wAFROC FOM scores of all readers with and without CAD were 0.746 (95% confidence interval [CI] 0.668–0.823) and 0.810 (95% CI 0.746–0.873), respectively. Thus, the increase in the wAFROC FOM score by the CAD software was 0.064. The mean wAFROC FOM score with CAD was significantly higher than without CAD (*P* = 0.007). Fig. 1 shows ROC curves with and without CAD. When stratified by the specialties of the readers, the mean wAFROC FOM scores for radiologists, pulmonologists, non-pulmonology physicians, and junior residents without CAD were 0.806 (95% CI 0.699–0.913), 0.817 (95% CI 0.714–0.920), 0.746 (95% CI 0.677–0.815) and 0.613 (95% CI 0.535–0.692), respectively. The wAFROC FOM scores increased with use of the CAD software to 0.835 (95% CI 0.765–0.904), 0.839 (95% CI 0.763–0.915), 0.815 (95% CI 0.747–0.884) and 0.749 (95% CI 0.6338–0.861), respectively. Thus, the increments were 0.029, 0.022, 0.069, and 0.136, respectively. The mean wAFROC FOM score of non-expert doctors (with < 6 years of experience) significantly improved with CAD from 0.680 (95% CI 0.586–0.773) to 0.779 (95% CI 0.705–0.853) (*P* = 0.011). The wAFROC FOM scores of experts (with ≥ 6 years of experience) also improved from 0.811 (95% CI 0.745–0.877) to 0.841 (95% CI 0.782–0.899) with CAD, but the difference was not significant (*P* = 0.12). The increments for each group were 0.099 and 0.030, respectively. Table 2 summarizes the results.

**Table 2:** Figure of merit scores for the detection of abnormal findings on chest radiographs with and without the computer-aided detection (CAD) software

	Without CAD	With CAD	Increment	*P* value
Total	0.746	0.810	0.064	0.023^a^
Observer group				
Radiologist	0.806	0.835	0.029	0.14
Pulmonologist	0.817	0.839	0.022	0.34
Physician	0.746	0.815	0.069	0.029^a^
Junior resident	0.619	0.749	0.136	0.078
Years of experience				
< 6	0.680	0.779	0.099	0.011^a^
≥ 6	0.811	0.841	0.030	0.12

^a^*P* < 0.05

### Analyses of factors influencing sensitivity and final diagnosis with CAD assistance

**Fig. 2:**
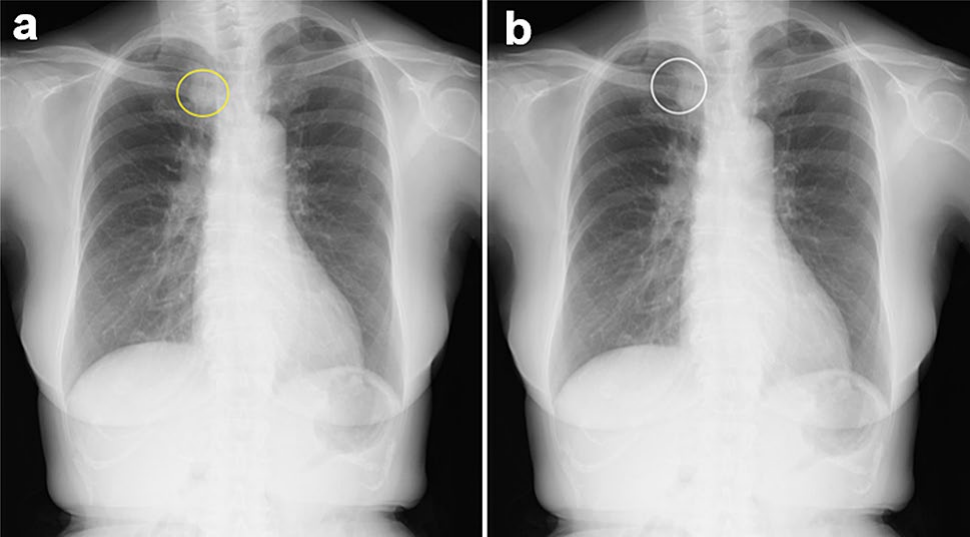
Examples of true-positive cases of pulmonary nodules. Four readers missed the lesion but corrected their decisions using the computer-aided detection (CAD) software. **a** The radiograph has a nodule at the right upper field, which is overlapped by the mediastinum. The yellow circle shows the ground truth. **b** The white circle shows the output of the CAD software. The software correctly detected the lesion

**Fig. 3:**
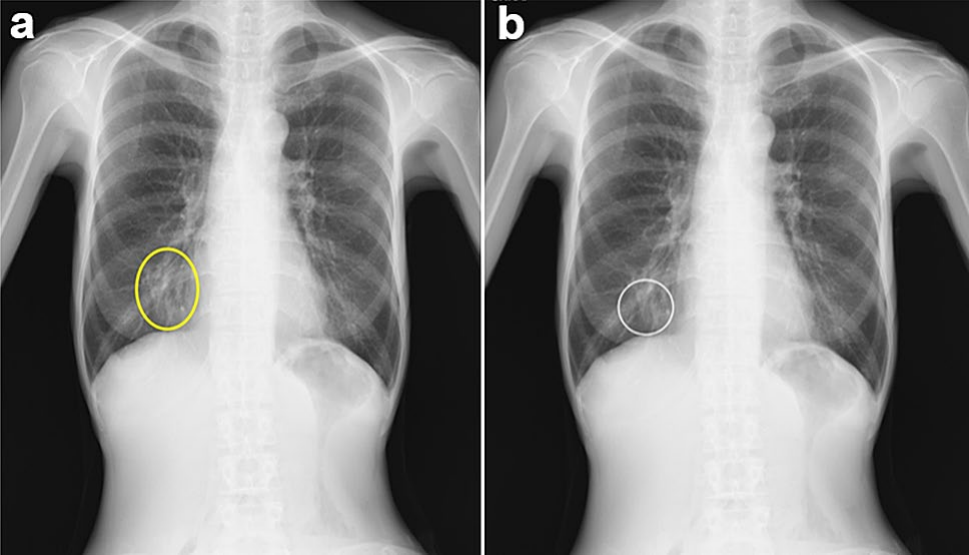
Example of true-positive cases of pulmonary consolidation. Five readers missed the lesion but corrected their decisions using computer-aided detection (CAD) software. **a** The radiograph shows consolidation in the right lower field. The yellow circle shows the ground truth. **b** The white circle shows the output of the CAD software. The software correctly detected the lesion

Non-experts and experts detected 66% (256/390) and 78% (305/390), respectively, of all abnormal lesions without using CAD. Table 3 shows the sensitivity of the CAD software and human readers, stratified by anatomic location and degree of subtlety of the lesion. Experts had higher sensitivity than non-expert doctors in all groups. The sensitivity of the CAD software for detecting obscure lesions (subtlety level 1, 2 or 3) was significantly lower than those for distinct lesions (subtlety level 4 or 5) [50% (10 of 20) vs 93% (42/45); *P* < 0.001]. The human readers initially missed 117 lesions without CAD. For 78 (67%) of these lesions, detection by the CAD software was accepted by each reader. Figures 2 and 3 show true-positive examples of pulmonary nodules and consolidation, which some readers missed without using CAD but acknowledged after using the software. Table 4 describes the accepted lesions, stratified by their characteristics. Of the initially missed lesions, 67% (55/82) and 66% (23/35) were corrected with CAD in the non-expert and expert groups, respectively. When stratified by the degree of subtlety, the adoption rate of obscure lesions and 50% (54/73) was significantly lower than that of distinct lesions [55% (24 of 44) vs 74% (54/73); *P* = 0.04]. The CAD software did not detect 13 lesions, and 12 of these were detected by at least one human reader.

**Table 3:** Characteristics of abnormal lesions detected by the computer-aided detection (CAD) software alone and by human readers alone in the observer-performance test

	CAD	Reader (< 6 years’ experience)	Reader (≥ 6 years’ experience)
Total	80 (52/65)	66 (256/390)	78 (305/390)
Side			
Right	78 (31/40)	61 (146/240)	75 (179/240)
Left	84 (21/25)	73 (110/150)	84 (126/150)
Location			
Upper	77 (10/13)	58 (45/78)	73 (57/78)
Middle	95 (18/19)	72 (82/114)	84 (96/114)
Lower	73 (24/33)	65 (129/198)	77 (152/198)
Lesion type			
Nodule (≤ 3 cm)	73 (11/15)	66 (59/90)	73 (66/90)
Mass (> 3 cm)	83 (15/18)	62 (67/108)	75 (81/108)
Consolidation	81 (26/32)	68 (130/192)	82 (158/192)
Degree of subtlety			
2	33 (3/9)	46 (25/54)	56 (30/54)
3	67 (7/11)	30 (20/66)	59 (39/66)
4	94 (16/17)	75 (76/102)	84 (86/102)
5	93 (26/28)	80 (135/168)	89 (150/168)

Data in parentheses are those used to calculate the percentages. Images with degree of subtlety 1 were not included in the dataset

**Table 4:** Adoption rate of abnormal lesions that were initially missed by readers but detected by computer-aided detection software

	Reader (< 6 years’ experience)	Reader (≥ 6 years’ experience)
Total	67 (55/82)	66 (23/35)
Side		
Right	63 (36/57)	58 (15/26)
Left	76 (19/25)	89 (8/9)
Location		
Upper	65 (15/23)	64 (7/11)
Middle	69 (20/29)	73 (11/15)
Lower	67 (20/30)	56 (5/9)
Lesion type		
Nodule (≤ 3 cm)	79 (11/14)	50 (4/8)
Mass (> 3 cm)	71 (22/31)	81 (13/16)
Consolidation	60 (22/37)	59 (6/11)
Degree of subtlety		
2	0 (0/3)	33 (1/3)
3	62 (18/29)	56 (5/9)
4	81 (17/21)	82 (9/11)
5	69 (20/29)	67 (8/12)

Data in parentheses are those used to calculate the percentages. Denominator is the sum of the number of lesions that were initially missed by readers. Images with degree of subtlety 1 were not included in the dataset

## Discussion

This study compared doctors’ performances in interpreting CRs with and without using CAD. The CAD software achieved about 80% sensitivity for detecting pulmonary nodules, masses, and consolidation on CRs in the standalone test. The observer-performance test showed that using the CAD software significantly increased the wAFROC FOM scores for these lesions.

Several studies have demonstrated that the assistance of deep learning-based algorithms yields higher detection performance than that achieved by human readers alone [11, 12]. The results of this study consistent with previous findings. Hwang et al. showed a significant improvement in the area under the curve for lesion-wise localization in various reader groups (from 0.781–0.907 to 0.873–0.938) [11]. Choi et al. reported that the assistance of a deep learning-based algorithm improved the FOM score from 0.843 to 0.911 [12]. However, these studies used algorithms developed from scratch for academic purposes.

This study used a CAD software package to demonstrate the utility of CAD. The FOM scores reported in previous studies were higher than those recorded in this study (0.746 to 0.810). However, the mean increments in FOM scores with CAD in the previous studies were 0.057 and 0.068, respectively, which were almost the same as those obtained in our study (0.064). Thus, while the lower FOM score in this study may be attributable to the difficulty of the dataset used, the degree of contribution of the software used is comparable to that of previous studies.

The increment in FOM scores by CAD was higher for non-pulmonology physicians and junior residents than for pulmonologists and radiologists. It was also higher for doctors with < 6 years of experience than for doctors with ≥ 6 years of experience. Thus, this study shows that CAD software is more useful for non-expert doctors than for expert doctors. In support of this findings, previous studies have also reported that CAD software was more beneficial for non-expert readers than expert readers [11, 12, 16].

In contrast, few studies have analyzed factors that influence readers’ performance and final diagnosis with the use of CAD. In our study, the CAD software yielded significantly less sensitive for detecting obscure lesions than for distinct lesions. The adoption rate of obscure lesions detected by the CAD software was also significantly lower than that of distinct lesions. These results revealed that detection of obscure lesions contributed less to the improvement of readers’ performance with CAD than detection of distinct lesions. Furthermore, adoption rate of CAD software detection for initially missed lesions by non-experts and experts were approximately the same (67% and 66%, respectively). Therefore, this study showed that CAD software was more effective for non-experts than experts because non-experts missed more distinct lesions than experts, and those lesions were detected by the CAD software.

The use of deep learning-based detection algorithms as second readers has already been described [11, 12, 17]. Such software packages can be adopted as second readers in daily practice, such as during medical checkups. The software automatically marks the regions where abnormal findings are suspected; thus, even non-expert doctors can recognize the lesions. Approximately 50% of doctors in Japan who read CRs for screening are not experienced readers [8]. Additionally, visual and mental fatigue caused by heavy workloads can increase the chances of perceptual errors [18]. The use of CAD software in institutions can therefore help to reduce misdiagnoses caused by these factors. However, the disadvantage of second-reader CAD is that it takes longer to read images with CAD than without CAD because of the necessity of two reading passes [19]. Therefore, some studies have highlighted the potential of using CAD software as a concurrent reader for CRs [11, 12]. On the other hand, the use of CAD software as a concurrent reader is associated with the risk that human readers may not pay attention to lesions that the software fails to detect. 20% of the abnormal findings in our study were not detected by the CAD software, and 92% of those lesions were detected by at least one reader. Using this software as a concurrent reader may lead to missing these lesions. To the best of our knowledge, no study has validated the effect of deep-learning-based algorithms on CRs as concurrent readers. Therefore, further study will be required to determine which reader type is more suitable in routine clinical practice.

This study has several limitations. First, validation was performed using small, designed datasets. In our study, 30% of the images in the observer-performance test showed pulmonary nodules/masses and consolidation. By contrast, one study reported that only 8% of the CRs taken for mandated health examinations showed any abnormal finding [20]. Thus, the prevalence of abnormal findings in this study was relatively higher than what is usually seen in routine practice. This may affect the adoption rate of CAD software detection. Second, CT was not used for ground truth labeling. Although the images were reviewed by three board-certified radiologists, some lesions might have been missed. Last, this study was conducted in accordance with the US Food and Drug Administration guideline, not the Japanese guidelines. Therefore, the performance could not be accurately compared with other products marketed in Japan.

In summary, this sequential evaluation study showed that the CAD software improved doctors’ performance in detecting nodules/masses and consolidation on CRs, particularly for non-expert doctors, by preventing doctors from missing distinct lesions rather than by helping them to detect obscure lesions. This software may prevent doctors from missing incidental lung abnormalities such as lung cancers, in clinical practice, due to inexperience and carelessness. Further prospective studies using multicenter data are required to validate the contribution of CAD software packages to clinical practice.
